# Perceptions vs. practices: academic integrity and actual ChatGPT use among EFL students

**DOI:** 10.3389/fpsyg.2026.1796737

**Published:** 2026-03-19

**Authors:** Reem Alsadoon

**Affiliations:** English Department, Imam Mohammad Ibn Saud Islamic University (IMSIU), Riyadh, Saudi Arabia

**Keywords:** academic integrity, ChatGPT, EFL writing, Perception, student behavior

## Abstract

This mixed-methods study explores how English as a Foreign Language (EFL) students and their teachers think about and actually use ChatGPT in academic writing, and how this relates to academic integrity. A unique contribution of the study is its use of observed behavioral data, capturing students' real-time interactions with ChatGPT rather than relying solely on self-reports. 60 female Saudi EFL students wrote a timed argumentative essay in a computer lab where they were allowed to use ChatGPT. Screen recordings captured all their prompts and AI outputs. These texts were later compared with students' final essays using copy ratio and Levenshtein distance to show how much they copied or revised. Students also completed pre- and post-task surveys about their ChatGPT use and integrity beliefs, and 20 teachers rated how acceptable eight ChatGPT functions were for academic writing. Short interviews were conducted with a sample of students. Findings showed that students viewed ChatGPT more positively than teachers and spent about one third of the writing time using it, mainly for editing and brainstorming. Cluster analysis identified three user types: constructive, hybrid, and substitutive, with about one-fifth of students relying heavily on AI text. Higher copying was linked to weaker integrity beliefs, while more revision and clearer policy awareness were linked to stronger ethical views. The study calls for clearer AI policies, process-based assessment, and practical AI literacy training.

## 1 Introduction

The emergence of generative artificial intelligence (GenAI) systems, most prominently ChatGPT, has introduced profound transformations to higher education. These systems can produce fluent, contextually appropriate texts, offer explanations, and generate linguistic feedback across a wide range of domains (Chan and Colloton, 2024). While such capabilities create opportunities for enhancing student learning, they simultaneously provoke serious debates about academic integrity (Bin-Nashwan et al., 2023). Students are increasingly using ChatGPT in their academic practices, which poses challenges for educators and institutions attempting to distinguish between legitimate pedagogical use and applications that compromise academic honesty and the authenticity of student performance (Cotton et al., 2024; Lo, 2023).

For English as a Foreign Language (EFL) learners, the implications of ChatGPT are particularly complex. On the one hand, access to instant feedback on grammar, vocabulary, and discourse enables students to enhance their writing skills and gain confidence in academic tasks (Xiao and Zhi, 2023). On the other hand, these same functionalities create opportunities for overreliance on AI-generated text, plagiarism, and

the misrepresentation of students' actual language proficiency. The possibility that learners may copy substantial portions of their academic work from ChatGPT can lead to invalid institutional assessments of student writing and hinder the development of autonomous communicative competence, which is central to language learning (Almanea, 2024; Lo et al., 2024).

Research to date has predominantly focused on attitudes and perceptions. Survey studies (e.g., Almanea, 2024; Bin-Nashwan et al., 2023; Khasawneh, 2024) suggest that educators are apprehensive about GenAI misuse, originality, critical thinking, and fairness in assessment (Werdiningsih and Marzuki Rusdin, 2024). Students, by contrast, often regard ChatGPT as a study assistant, language learning tutor, and language editor (Xiao and Zhi, 2023). (Almanea 2024) notes that students may underreport practices that could be construed as misconduct, and teachers' perceptions are often shaped by institutional norms and ethical frameworks (Denkin, 2024; Hasan et al., 2025). However, despite this growing body of work, a critical gap remains between stakeholders' reported perceptions of ChatGPT and students' actual practices in authentic writing contexts.

This gap is particularly consequential in EFL contexts, where the stakes extend beyond individual assignments to the long-term development of linguistic proficiency. Excessive reliance on AI tools may yield short-term academic success while masking persistent deficits in language competence (Zhai et al., 2024). Alsaedi's (2024) systematic review highlights overreliance on AI as a key challenge that may undermine ESL/EFL learners' development of original expression, critical thinking, and internal writing skills. Understanding EFL learners' real practices with GenAI is therefore critical for informing effective policies and pedagogical responses to academic integrity concerns.

To address this gap, the present study adopts a lab-controlled design that directly observes students' real-time engagement with ChatGPT during academic writing tasks. Screen recordings of student–AI interactions provide empirical evidence of actual usage patterns. These behavioral data are triangulated with students' self-reported perceptions and teachers' evaluations of appropriate AI use, offering a more comprehensive account of how GenAI is used in practice. By combining observed behavior with perception data, the study advances prior research on AI and academic integrity in EFL contexts.

Ultimately, this research contributes to ongoing global debates on the ethical integration of AI in higher education. Its findings inform institutional policies aimed at upholding fairness and accountability and provide educators with evidence-based insights for guiding students toward constructive and responsible uses of AI. In doing so, the study offers practical implications for language learning, technology integration, and academic ethics.

## 2 Literature review

This section outlines the theoretical framework underpinning the study and synthesizes previous research to clarify how the present study addresses unresolved gaps in the literature.

### 2.1 Theoretical framework

The theoretical framework for this study encompasses principles from Sociocultural Theory (SCT) and Academic Integrity Theory, which are used to discuss the educational and ethical implications of GenAI use by EFL students. These frameworks provide complementary perspectives on how learners interact with technological tools like ChatGPT and how such interactions influence language learning, autonomy, and integrity in academic contexts.

SCT emphasizes learning as a socially mediated process where knowledge is constructed through interaction, collaboration, and culture (Vygotsky, 1978). In the case of language learning, L2 learning occurs through mediated interaction where learners acquire linguistic input via scaffolding provided by more capable peers, teachers, or tools (Kim, 2025). This kind of interaction is referred to as Zone of Proximal Development (ZPD) by (Vygotsky 1978). In this study context, ChatGPT functions as a mediational tool assisting language learners in improving their language production through instant feedback, expert language knowledge, and autonomy. When learners interact with ChatGPT to refine their grammar or structure their writing, they engage in an interaction similar to peer or teacher mediation. The AI tool can therefore extend the ZPD by enabling independent practice. However, it is important to be careful with the risks of over-scaffolding with GenAI, where learners depend excessively on external mediation and fail to internalize skills (Baskara, 2023). According to (Lo et al. 2024), overreliance on AI tools occurs when EFL/ESL learners depend on ChatGPT to generate entire texts or assignments resulting in hindering the development of autonomous linguistic competence.

While SCT explains the cognitive and behavioral dimensions, Academic Integrity Theory provides the ethical framework through which the use of GenAI must be investigated. In higher education, academic integrity is based on a set of principles: honesty, trust, fairness, responsibility, and respect (McCabe et al., 2001). According to (Bretag 2013), integrity is not merely compliance with institutional policy but a reflection of individuals' moral reasoning and ethical responsibility. With this theoretical framework in mind, the use of ChatGPT represents an ethical process where learners must balance the need for efficiency with the value of authenticity. (Bretag 2013) argues that the behaviors of teachers and students are shaped by the moral and policy environment of their academic organizations. Institutions that fail to provide clear guidelines for AI use may inadvertently foster uncertainty and misconduct (Hasan et al., 2025). Hence, understanding the ethical context of ChatGPT use is essential for interpreting how learners act and for designing better interventions.

Rather than functioning as parallel explanations, SCT and Academic Integrity Theory jointly inform the study's variables and analytical focus. SCT guides the operationalization of behavioral variables, such as observed patterns of AI interaction, revision behavior, and degrees of reliance on ChatGPT as a mediational tool within the ZPD. Academic Integrity Theory, in turn, informs the ethical dimension of the study, shaping the examination of integrity beliefs, perceptions of acceptable AI use, and alignment between attitudes and practices. By integrating these perspectives, the framework links observable writing behaviors with underlying ethical orientations, allowing the analysis to capture both how learners use ChatGPT and how they morally interpret that use.

By integrating these theoretical strands, the framework positions GenAI use as both a sociocultural and ethical phenomenon. From SCT, ChatGPT is a mediational tool that can support or hinder learners' ZPD depending on engagement quality. From Academic Integrity Theory, AI use reflects ethical decision-making shaped by individual attitudes and institutional context. Together, this integration provides a unified lens for interpreting the study's mixed-methods design, where behavioral data (e.g., screen-recorded interactions and revision patterns) are analyzed alongside perception data to examine the convergence or divergence between practice and ethical judgment.

### 2.2 GenAI, education, and academic integrity

The rise of GenAI systems like ChatGPT has sparked a debate about their educational benefits and ethical challenges in higher education (Chan and Colloton, 2024). GenAI systems can generate fluent and coherent texts and give instant feedback across different domains, which presents affordances for teaching and learning, but also challenges to academic integrity (Cotton et al., 2024). GenAI has a double role as both a valuable teaching tool and a source of academic risk. It can enhance learning through personalized instant feedback, but it can also harm academic integrity when misused for ghostwriting or overreliance (Ibrahim et al., 2023).

Researchers emphasize that in AI-driven contexts, detection tools alone are insufficient to ensure academic integrity, especially given their current limitations in accuracy (Almanea, 2024; H. Ibrahim et al., 2023; Werdiningsih and Marzuki Rusdin, 2024). Obviously, there is a need for new assessment designs, stronger AI literacy, clear institutional policies, and teaching models that focus on process, guidance, and student involvement (Cotton et al., 2024; Sullivan et al., 2023).

These challenges are greater in language learning, where writing and speaking are central for developing language proficiency. If students depend too much on AI, teachers may misread their skills, and learners may struggle to build their metalinguistic competencies and autonomous learning (Alsaedi, 2024; Lo et al., 2024).

Collectively, these studies establish the dual pedagogical and ethical nature of GenAI but are largely grounded in conceptual discussions or perception-based evidence rather than direct observation of learner behavior.

### 2.3 The role of ChatGPT in EFL/ESL settings

Recent research in EFL and ESL contexts highlights both the pedagogical affordances and ethical tensions associated with ChatGPT integration. Scholars widely agree that ChatGPT can support writing development by assisting with drafting, grammatical revision, lexical expansion, and fluency (Almanea, 2024; Xiao and Zhi, 2023). Intervention studies have shown that using ChatGPT as a formative feedback tool can enhance writing accuracy, organization, and learner engagement while reducing anxiety (Khampusaen, 2025; Mahapatra, 2024). Similarly, (Mizumoto et al. 2024) demonstrated that AI-generated texts exhibit higher lexical diversity and syntactic complexity compared to student writing, underscoring both the pedagogical potential and integrity concerns associated with AI-assisted production. Meta-analytic evidence further indicates that GenAI can improve motivation, feedback access, and learner autonomy, while raising concerns about reduced critical thinking, unequal access, and ethical misuse (Ibrahim and Kirkpatrick, 2024).

Beyond writing, emerging research suggests that AI-mediated interactions may support speaking development and vocabulary learning through scaffolded dialogue and adaptive feedback (Fathi et al., 2024; Abdelhalim and Alsehibany, 2025). Collectively, these studies highlight ChatGPT's capacity to function as a supplementary instructional tool, particularly for lower-proficiency learners, while simultaneously emphasizing the risks of overreliance and diminished learner autonomy.

Alongside affordance-focused work, a growing body of research examines learner and instructor perceptions of ChatGPT. Teacher-focused studies report mixed attitudes, with some educators valuing ChatGPT's efficiency and responsiveness, while others express concerns about its effects on critical thinking, research skills, and academic honesty (Jeon and Lee, 2023; Kohnke et al., 2023; Mohamed, 2024). Learner-focused studies generally report positive perceptions, linking ChatGPT use to increased motivation, engagement, and perceived writing improvement (Teng, 2024; Kivrak, 2024; Othman, 2025). However, perception studies also highlight ambivalence. For instance, (Almanea 2024) found that students acknowledged potential misuse, including hidden AI use or plagiarism, while teachers expressed uncertainty about appropriate boundaries for AI integration. Similarly, (Slamet 2024) reported that both instructors and students recognized pedagogical benefits but raised concerns about dependence and ethical ambiguity.

Taken together, this literature suggests that while ChatGPT is widely perceived as pedagogically valuable, perceptions remain divided and context-dependent. Importantly, several studies note that students may underreport AI use due to institutional or ethical pressures (Almanea, 2024), and that teachers' attitudes are shaped by local norms and enforcement environments (Hasan et al., 2025). This reliance on perception-based data highlights a persistent limitation in the literature.

Despite rapid growth in this field, empirical work capturing learners' real-time engagement with ChatGPT remains scarce. Much of the existing research relies on surveys and interviews, which may underestimate the extent and nature of AI use. Few studies document interaction logs, screen recordings, or revision trajectories that reveal how learners operationalize ChatGPT during authentic writing tasks. Without behavioral evidence, it remains difficult to determine whether reported attitudes align with actual practices. Addressing this perception–practice gap is therefore essential for advancing research on GenAI and academic integrity in language learning.

## 3 Research questions

The present study investigates EFL students' real-time use of ChatGPT during academic writing and examines how observed behaviors align with learner and teacher perceptions of academic integrity. Specifically, the study addresses the following analytical aims:

1. To examine how EFL students use ChatGPT during a controlled academic writing task, including the types and frequencies of functional uses (e.g., brainstorming, drafting, editing).
2. To analyze the extent to which students' observed ChatGPT use (e.g., copy ratio and revision distance) aligns with their self-reported perceptions of academic integrity.
3. To compare student and teacher perceptions of acceptable and unacceptable ChatGPT use in academic writing.
4. To identify perceptual and behavioral gaps between observed student practices and stakeholder beliefs regarding ethical AI use in EFL writing contexts.

## 4 Method

### 4.1 Research design

This study employed a mixed-methods design, integrating quantitative and qualitative data, to investigate EFL students' actual and perceived use of ChatGPT in academic writing, as well as teachers' perceptions of academic integrity. A mixed-methods approach was selected to capture both observable behavioral patterns and underlying ethical perceptions, aligning with the study's integrated sociocultural and academic integrity framework. The present study adopted a controlled laboratory experiment to capture authentic behavioral data, while post-task surveys and semi-structured interviews triangulated participants' perceptions and practices. A lab-based design was chosen to ensure ecological validity while enabling precise observation of real-time AI interaction, addressing limitations of prior perception-only studies. The design thus enabled examination of both the extent and nature of ChatGPT use and the perceptual gap between teachers and students consistent with mixed-methods triangulation approaches (Hashemi and Babaii, 2013).

### 4.2 Participants

Two participant groups were recruited from the College of Languages and Translation, Imam Mohammed Ibn Saud Islamic University, in Riyadh, Saudi Arabia. The first group consisted of 60 female students enrolled in the Level 4 Essay Writing II course (ENG 1215). This cohort was selected because it represents an intermediate academic writing stage in which students possess sufficient linguistic proficiency to engage in extended writing while still relying on external support tools, making it an appropriate context for examining AI-mediated scaffolding. The second group included 20 female teachers who taught the same course or other similar writing courses in the same college. The sample was drawn from a gender-homogeneous institutional context, reflecting the structure of the local higher education system and participant accessibility. This enabled consistency in instructional norms and assessment expectations while minimizing contextual variability during behavioral observation. Participation was voluntary, and all participants were provided with written information about the study and signed informed consent forms. Based on demographic data from a pre-task survey (see Appendix A), all the participants reported using GenAI tools for learning and writing, primarily ChatGPT. Furthermore, 77.2% of the participants reported using these tools weekly. However, only 34.7% confirmed receiving formal guidance on responsible AI use.

### 4.3 Materials and instruments

#### 4.3.1 Writing task

Students completed a 45-min argumentative essay (300–350 words) modeled on regular course assessments. The argumentative genre was selected because it requires cognitive effort, critical thinking, and careful linguistic structuring, making it well suited for observing multiple forms of AI mediation. They were allowed to consult online resources, including ChatGPT. Sessions took place in a computer laboratory equipped with clean system profiles. A window-restricted screen recorder (OBS Studio) captured only browser activity, and a lightweight logging extension recorded time stamps, active-tab data, and copy-paste events. No audio, webcam, or personal desktop data were collected.

#### 4.3.2 Questionnaires

Three validated online questionnaires were administered in this study. The first, a pre-task student questionnaire (see Appendix A), elicited students' prior experience with ChatGPT, frequency of AI use, and perceptions of acceptable vs. unethical practices in academic writing. It contained six sections assessing background variables, typical use, integrity boundaries, ethical stance, policy awareness, and pre-task confidence. All items used Likert-type scales, providing baseline data on learners' attitudes and readiness before participating in the controlled writing experiment.

The second, a post-task student questionnaire (see Appendix B), measured the perceived acceptability of ChatGPT use across eight functions—brainstorming, outlining, drafting, editing, translation, paraphrasing, summarizing, and referencing—using a 5-point Likert scale. Administering this instrument after the lab session enabled comparison between reported beliefs and observed behaviors. Using 25 fully closed-ended items, it gathered students' self-reported behaviors, reflections, and integrity perceptions after the lab writing session.

The third, a teacher questionnaire (see Appendix C), examined instructors' perceptions of ChatGPT use in EFL writing and its implications for academic integrity, pedagogy, and policy. Comprising 24 quantitative items, it explored teachers' acceptance of different ChatGPT functions, beliefs about risks and benefits, institutional policy awareness, and preferred disciplinary approaches. All items were reviewed by three applied linguistics experts and piloted with ten participants, yielding a Cronbach's alpha of 0.86, indicating high reliability.

#### 4.3.3 Recorded data and coding

All ChatGPT prompts observed in the recordings were classified using a structured prompt function codebook (see Appendix D), which included eight categories: Brainstorming (BRAIN), Outlining (OUTL), Drafting (DRAFT-minor/DRAFT-major), Editing (EDIT), Translation (TRANS), Paraphrasing (PARA), Summarizing (SUMM), and Citation (CITE). Coders entered classifications in a standardized Excel sheet. Each code in the manual was clearly defined and illustrated with examples, along with rules to resolve ambiguity. For instance, if a prompt combined two functions (e.g., “translate and paraphrase”), both codes were marked; if unclear, coders provided clarification in the comments section. Coders were instructed to focus on the primary purpose of each prompt. To ensure reliability, two independent coders analyzed the same sample, and interrater agreement was assessed with a target of κ ≥ 0.75, in line with established qualitative coding standards (Awan et al., 2023).

#### 4.3.4 Text-comparison metrics

To objectively assess the extent of students' reliance on ChatGPT, the study employed text-diffing analysis comparing each student's final submission with the corresponding ChatGPT outputs recorded during the lab session (Huang et al., 2011). These metrics were selected to quantify observable dependence on AI-generated language, providing a behavioral proxy for autonomy vs. substitution.

The study used two comparison metrics: the copy ratio and the Levenshtein edit distance. The copy ratio represented the proportion of textual material directly matching ChatGPT-generated text (Li, 2025). The second metric, Levenshtein distance, quantified the extent of revision required to transform AI-generated text into the final submission (Huang et al., 2011; Van der Loo, 2014). Together, these metrics enabled fine-grained measurement of behavioral reliance on AI, complementing self-reported integrity perceptions.

The computations were conducted in R (RStudio) using stringdist and quanteda packages. The computations employed established text-analysis packages: stringdist (Van der Loo, 2014) for Levenshtein distance and quanteda (Benoit et al., 2018) for token-based similarity (see Appendix E). Token-based similarity measures have been widely used in computational text comparison and authorship analysis (Benoit et al., 2018). The outputs were exported as numeric variables and analyzed in SPSS for correlations with survey-based integrity perceptions.

#### 4.3.5 Interviews

To gain qualitative insight into learners' ethical reasoning and decision-making, semi-structured interviews (see Appendix F) were conducted with a purposive subsample of 10 students after the writing task. Interviews explored experiences with ChatGPT, perceived advantages and concerns, and views on acceptable vs. inappropriate uses in academic contexts. Each interview lasted 5–10 min, was audio-recorded with consent, and transcribed for thematic analysis. Interviews provided interpretive depth by exploring how students justified their AI use, thereby contextualizing quantitative perception–behavior divergences.

### 4.4 Procedure

The data collection process included six main stages. First, in the orientation and consent stage, participants were informed about the study's purpose, privacy protections, and data-recording process before giving written consent. Second, a pre-task survey collected information about students' previous experience with AI tools and their views on ethical limits. Third, during the writing session, each student wrote an essay individually while being observed, and all ChatGPT interactions and writing activities were recorded. Fourth, a post-task survey asked students to reflect on the functions they used, why they used them, and how acceptable they found them. Fifth, follow-up interviews were held with ten volunteers who explained their decision-making during the task. Finally, teachers completed the online questionnaire about their perceptions. This sequential design mirrored the study's analytical logic, beginning with behavioral observation and subsequently contextualizing observed patterns through perception data.

### 4.5 Data analysis

Quantitatively, descriptive statistics summarized ChatGPT usage patterns and time spent on tasks. Behavioral measures included number of prompts, time using ChatGPT, copy ratio, and edit distance, while perceptual measures included acceptability and integrity-belief scores. Data were screened for normality and variance assumptions prior to parametric testing. Independent-samples *t*-tests and ANOVAs compared group differences, Pearson's r examined perception–behavior correlations, and multiple regression identified predictors of integrity beliefs. Inter-coder reliability for prompt classification was high (Cohen's κ = 0.81).

Analytic choices were guided by the study's mixed-methods framework, enabling triangulation between behavioral indicators and ethical perceptions (Hashemi and Babaii, 2013). Qualitative interview data were analyzed thematically using inductive coding and compared with quantitative results to explain divergences between reported and observed integrity practices (Awan et al., 2023).

### 4.6 Ethical considerations

All procedures adhered to institutional and international ethical standards. Screen recordings were limited strictly to browser windows; no personal data was captured. Participants were anonymized with numeric identifiers. Raw recordings were deleted after coding, and only de-identified text and coded variables were retained on encrypted drives. Data were used exclusively for research and never for disciplinary purposes. Participation was voluntary, with the right to withdraw at any time.

## 5 Analysis

This mixed-methods investigation examined how EFL students and teachers perceive and actually use ChatGPT in academic writing, focusing on integrity-related behaviors. Quantitative results from surveys, screen-recordings, and text-comparison metrics were triangulated with interview data. All quantitative findings are reported with corresponding descriptive and inferential statistics, with tables and figures referenced to ensure analytical transparency. Qualitative findings are presented thematically in alignment with each research question, with representative quotations used to contextualize the quantitative results.

### 5.1 RQ1: actual behavioral use during controlled writing

To examine how students used ChatGPT during the controlled writing task, behavioral data were extracted from screen recordings and prompt logs. Analysis of 512 recorded prompts (8.5 per student) showed that learners spent about one-third of the 45-min session using ChatGPT (*M* = 14.8 min, SD = 6.3), indicating substantial integration of AI support during the writing process (see Table 1). The Prompts were then coded into the eight predefined functional categories. Frequency of functional use is calculated as presented in Table 2. The most frequent functions were editing (22 %) and brainstorming (20 %), followed by outlining (12 %), paraphrasing (12 %), translation (9 %), and drafting (16 % combined), suggesting that students primarily used ChatGPT for refinement and generating ideas rather than full-text generation.

**Table 1:** Time-on task measures.

**Measure**	***M***	**SD**	**Range**
Total time in ChatGPT (min)	14.8	6.3	5–32
Total writing session (min)	44.9	2.1	40–49
% time with ChatGPT active	33 %	11	10–57

**Table 2:** Frequency of functional use.

**Function**	**Mean prompts per student**	**% of all prompts**	**Typical length of prompt (Words)**
Brainstorming (BRAIN)	1.7 (0.9)	20 %	11.2
Outlining (OUTL)	1.0 (0.7)	12 %	14.6
Drafting minor (DRAFT-m)	0.9 (0.8)	11 %	18.1
Drafting major (DRAFT-M)	0.4 (0.5)	5 %	24.3
Editing (EDIT)	1.9 (0.8)	22 %	13.8
Paraphrasing (PARA)	1.0 (0.6)	12 %	15.5
Translation (TRANS)	0.8 (0.6)	9 %	12.7
Summarizing (SUMM) + citation (CITE)	0.8 (0.5)	9 %	16.9

The pattern of use was analyzed using cluster analysis, which produced three behavioral profiles. The first group, constructive users (42% of participants), used ChatGPT mainly for idea generation and editing, with minimal copying. The second group, hybrid users (38%), combined editing and translation with a moderate level of copying. The third group, substitutive users (20%), relied heavily on ChatGPT for drafting and translation, showing substantial copying behavior.

**Figure 1:**
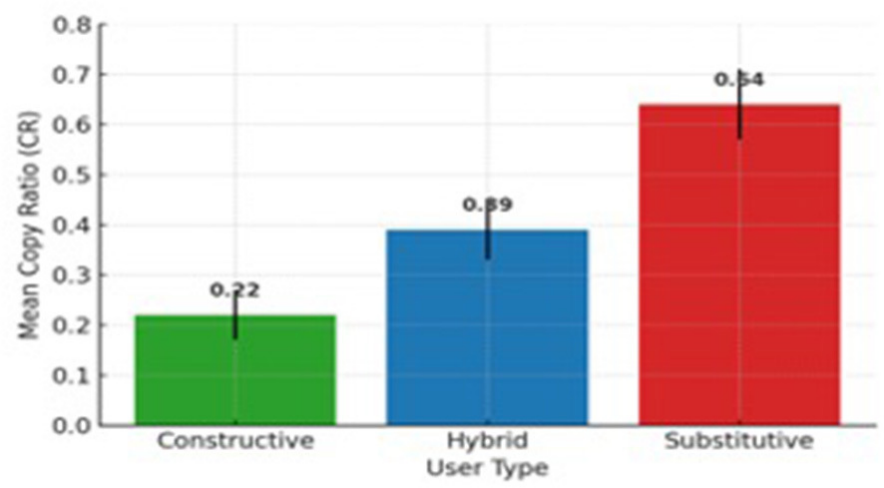
Comparison of mean Copy Ratios (CR) among the three user types.

Text-comparison metrics confirmed these patterns. The average copy ratio was 0.36 (SD = 0.18) and normalized Levenshtein distance 0.48 (SD = 0.17). Using text comparison calculations in R studio (i.e., the quanteda and stringdist) to measure lexical overlap and similarities. Constructive users showed the lowest overlap (CR ≈ 0.22) and greatest revision, while substitutive users showed the highest overlap (CR ≈ 0.64). Figure 1 demonstrates the difference in mean Copy Ratio (CR) across the three user groups, highlighting the higher textual overlap among Substitutive users (CR = 0.64) compared to Constructive (CR = 0.22) and Hybrid (CR = 0.39) groups. Furthermore, ANOVA was carried out revealing a significant between-group differences, [*F*_(2, 57)_ = 28.9, *p* < .001, η^2^ =0.50], representing a large effect size and confirming meaningful behavioral divergence (see Table 3).

**Table 3:** One-way ANOVA for copy ratio (CR) by user type.

**Source**	**SS**	**df**	**MS**	***F***	***P***	**η^2^**
Between groups	1.65	2	0.825	28.90	< 0.001	0.50
Within Groups	1.63	57	0.029			
Total	3.28	59				

These behavioral results show that most students used ChatGPT constructively for feedback and refinement, while about 20% relied on it substitutively, generating large portions of their text. Qualitative findings from the interviews support these patterns. Several students described using ChatGPT for efficiency and clarity, as one participant noted, “I only used it to polish sentences”. Others relied on it for language support, saying, for example, “I checked translations of Arabic ideas” and “Using translation can clarify concepts”. However, some participants admitted feeling tempted to copy: “When time ran out, I copied a full paragraph,” and “At first, I just wanted ideas, but when I saw how well it wrote, I ended up pasting more than I planned”. Overall, these findings highlight both the practical benefits of ChatGPT and ongoing uncertainty about acceptable boundaries in its use. Together, the converging quantitative and qualitative evidence indicates heterogeneous patterns of AI mediation ranging from scaffolding to substitution.

### 5.2 RQ2: relationship between actual use and integrity perceptions

To address this question, behavioral variables (i.e., CR and LD) and perceptual variable (i.e., IBI) are tested through correlation. Descriptive analysis as presented in Table 4 revealed that students generally viewed ChatGPT use as ethically acceptable (IBI ≈ 3.9), despite moderate levels of observed textual overlap.

**Table 4:** Descriptive analysis for CR, LD, and IBI.

**Variable**	***M***	**SD**	**Range**
Copy ratio (CR)	0.36	0.18	0.05–0.82
Levenshtein distance (LD)	0.48	0.17	0.15–0.79
Integrity-belief index (IBI)	3.87	0.48	2.8–4.8

**Figure 2:**
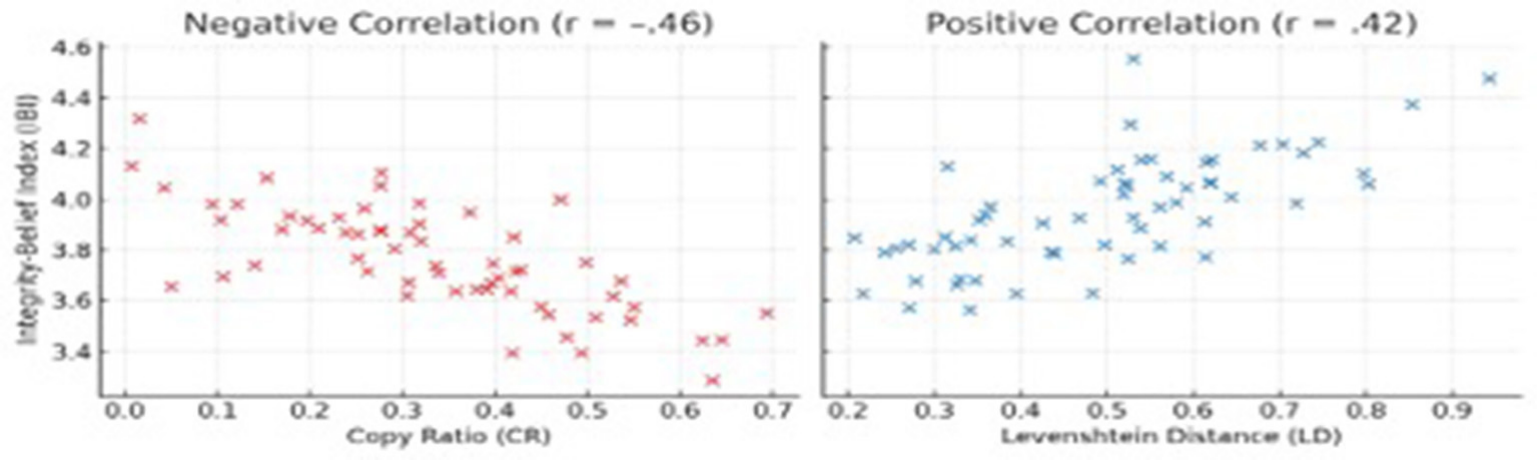
Scatterplots showing correlations between behavioral measures and integrity beliefs.

Correlation test revealed that CR correlated negatively with IBI scores (*r* = –.46, *p* < 0.01), whereas LD correlated positively (*r* = 0.42, *p* < .01), indicating moderate associations between behavioral reliance and ethical orientation (see Figure 2). Table 5 presents the multiple regression analysis predicting students' IBI. The model was significant, [*F*_(4, 55)_ = 8.67, *p* < 0.001, explaining 39% of the variance in integrity beliefs. Higher copying predicted weaker integrity orientation (β = −0.39, *p* = 0.001), whereas greater revision effort (Levenshtein distance) and stronger policy awareness predicted higher ethical awareness (β = 0.28, *p* = 0.037; β = 0.24, *p* = 0.047, respectively). Frequency of AI use showed a marginal positive trend (β = 0.20, *p* = 0.055).

**Table 5:** Multiple regression predicting IBI.

**Predictor**	**β**	***t***	***p***	**VIF**
Copy ratio	−0.39	−3.37	0.001	1.84
Levenshtein distance	+ 0.28	2.14	0.037	1.77
Frequency of AI use	+ 0.20	1.96	0.055	1.29
Policy awareness	+ 0.24	2.03	0.047	1.36

Group comparison confirmed the trend: high-dependence students (CR ≥ 0.35) reported lower integrity scores (*M* = 3.58) than low-dependence students (*M* = 4.06), *t* (58) = 4.17, *p* < 0.001, *d* = 0.91, indicating a large between-group difference (see Table 6). Students were grouped by their CR, which reflects the proportion of ChatGPT text reused, using the median value (0.35) as a cutoff. The independent-samples *t*-test as shown in Table 6 revealed that higher overlap with ChatGPT was linked to lower integrity beliefs, indicating a strong negative relationship between AI dependence and ethical orientation.

**Table 6:** Independent t-Test Comparing Integrity Scores by ChatGPT Dependence.

**Group**	***n***	***M***	**SD**	***t*(58)**	***p***	***d***	**95 % CI for mean difference**
Low-dependence (CR < 0.35)	30	4.06	0.48				
High-dependence (CR ≥0.35)	30	3.58	0.55	4.17	< 0.001	0.91	[0.25, 0.70]

Qualitative evidence mirrored this: students rationalized partial reuse as legitimate (“I only changed some words”) but those emphasizing revision saw ChatGPT as a scaffold (“It helps me notice my grammar mistakes”). Overall, the data show a behavior–belief mismatch: self-reported ethical awareness does not always translate into ethical practice.

### 5.3 RQ3: perceptions of ChatGPT use and academic integrity

To address this question, Students' acceptability of the eight ChatGPT functions (e.g., brainstorming, outlining, drafting, and editing) was measured using the post-task questionnaire. A composite Integrity-Belief Index (IBI) was calculated as the mean of 25 items, showing excellent reliability (α = 0.88). The teacher questionnaire included 24 items assessing perceptions of ChatGPT's ethical use and policy alignment, also demonstrating high reliability (α = 0.91).

As compared through independent *t*-test, students expressed generally positive views of ChatGPT as a learning aid (*M* = 3.87, SD = 0.48), whereas teachers reported more cautious attitudes (M = 3.25, SD = 0.55), t (78) = 5.13, *p* < 0.001, *d* = 1.15, indicating a large effect size (see Tables 7, 8).

**Table 7:** Descriptive statistics of students' and teachers' integrity-belief scores.

**Group**	***N***	**Mean**	**Std. deviation**	**Std. error mean**
Student	60	3.87	0.48	0.06
Teacher	20	3.25	0.55	0.12

**Table 8:** Comparison of students' and teachers' integrity-belief scores.

**Variable**	***t***	**df**	**Sig. (2-tailed)**	**Mean difference**	**Cohen's d**
IBI_Mean	5.13	78	< 0.001	0.62	1.15

**Figure 3:**
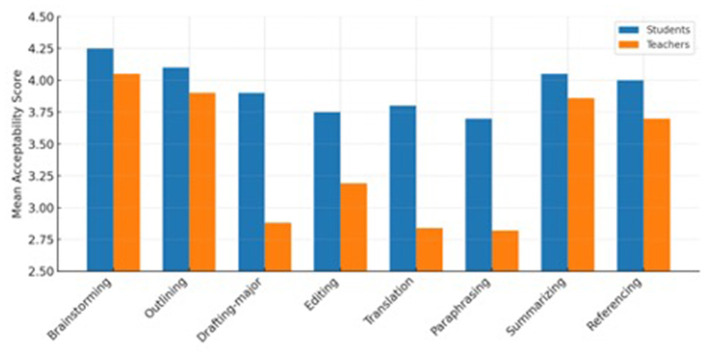
Mean acceptability ratings for eight ChatGPT functions by group.

A mixed-design ANOVA with Group (student vs. teacher) as the between-subjects factor and Function (8 levels) as the within-subjects factor revealed a significant main effect of Group, [*F*_(1, 78)_ = 21.6, *p* < 0.001, η*p*^2^ = 0.22], indicating that teachers were generally stricter in their evaluations. The Function × Group interaction was also significant, [*F*_(7, 546)_ = 9.4, *p* < .001, η*p*^2^ = 0.11], showing that group differences varied across functions. *Post hoc* comparisons (Bonferroni-adjusted) indicated that teachers rated drafting, translation, and paraphrasing as significantly less acceptable than students, while both groups viewed brainstorming and outlining as legitimate uses of ChatGPT as presented in Figure 3.

Further, regression analysis is carried out to test the relationships between major themes and IBI. The results of these multiple regression as presented in Table 9 indicated that students' frequency of ChatGPT use (β = 0.41, *p* = 0.001) and AI-literacy awareness (β = 0.28, *p* = 0.03) predicted stronger acceptance of its use, explaining 34 % of variance in integrity-belief scores (*R*^2^ = 0.34).

**Table 9:** Multiple regression predicting integrity-belief index (IBI).

**Predictor**	**β**	***t***	***p***	**95% CI (β)**	**VIF**
Frequency of ChatGPT use	0.41	3.49	0.001	[0.17, 0.64]	1.58
AI-literacy awareness	0.28	2.21	0.030	[0.03, 0.52]	1.42
Plagiarism awareness	−0.19	−1.64	0.107	[−0.42, 0.04]	1.25
Policy awareness	0.24	2.03	0.047	[0.01, 0.47]	1.36

Interview data reinforced these findings: most students described ChatGPT as “a study assistant, not cheating,” though several admitted uncertainty about institutional expectations. As one participant explained, “I'm not sure what the policy actually says about using ChatGPT—some teachers say it's fine for grammar, others say not at all.” Another student reflected, ‘It's confusing because we never received clear rules; I use it only when I think it won't cause trouble.'. Overall, the results show a perception gap: students see ChatGPT as a practical tool, while teachers take a cautious view focused on assessment fairness. Overall, the results demonstrate a clear perception gap between students and teachers.

### 5.4 RQ4: perceptual and behavioral gaps between students and teachers

**Figure 4:**
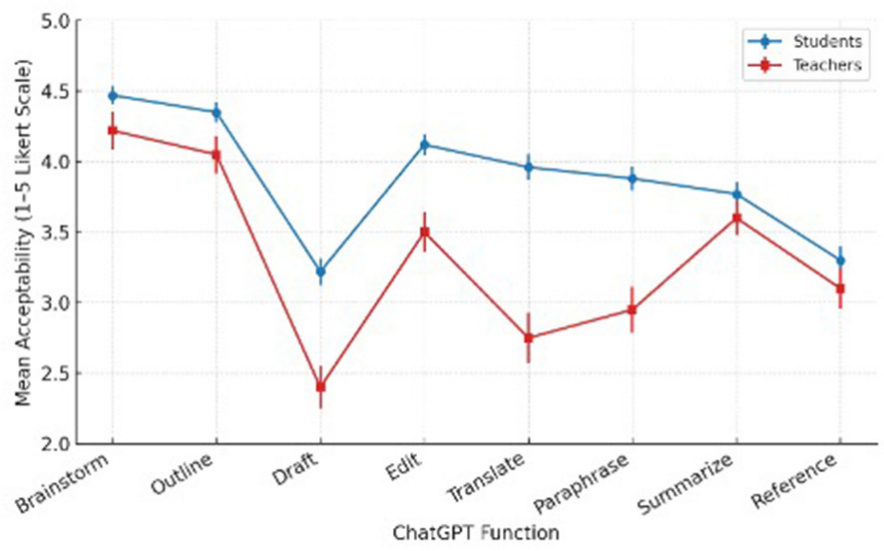
Mean acceptability ratings of eight ChatGPT functions among students and teachers.

The main variables considered for the analysis of this question are teachers vs. students based on their acceptability rating of the eight functions. For this reason, a mixed ANOVA test was carried out revealing a significant group effect, [*F*_(1, 78)_ = 21.6, *p* < 0.001, ηp^2^ = 0.22]. It confirms that teachers consistently applied stricter standards. Interaction effects across functions were significant, [*F*_(7, 546)_ = 9.4, *p* < 0.001, η*p*^2^ = 0.11. The largest discrepancies concerned drafting, translation, and paraphrasing (all *d* > 1). Figure 4 illustrates large gaps in drafting, translation, and paraphrasing, where teachers rated these functions as far less acceptable than students. It also shows a moderate gap in editing and minimal or no differences in brainstorming, outlining, summarizing, and referencing.

**Figure 5:**
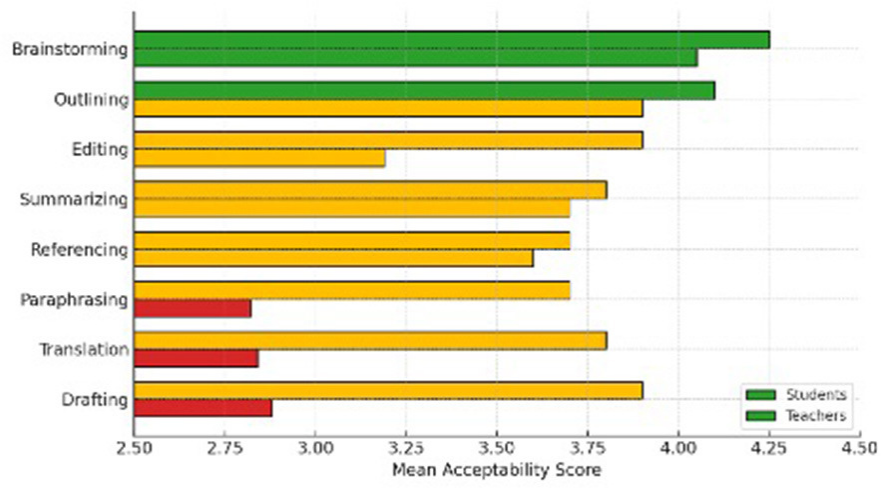
Comparison of students' and teachers' acceptability ratings across ChatGPT functions in EFL writing.

Rank-order agreement is also tested on what is most acceptable for teachers vs. students. Kendall's *W* = 0.48, *p* = 0.012 indicated moderate agreement on permissible uses (brainstorming, outlining, editing). The key disagreement is that teachers rank translation and drafting-major near the bottom; students place them mid-pack. Figure 5 compares how students (left bars) and teachers (right bars) rated each ChatGPT function. Teachers rated drafting, translation, and paraphrasing lower, indicating greater ethical caution, while both groups aligned on brainstorming and outlining.

Importantly, students' acceptability-gap scores (i.e., the link between behavior and perception) for drafting showed a significant positive correlation with CR (*r* = 0.49, *p* < 0.001) as presented in Table 10, indicating that students who viewed high-risk functions as more acceptable were also more likely to reuse ChatGPT-generated text. Qualitative results revealed contrasting moral logics between the two groups. Questionnaire responses showed that teachers emphasized authorship and fairness, viewing ChatGPT use beyond minor editing as a threat to academic integrity. In contrast, students explained their use of ChatGPT as a way to get language help and save time. Several interviewees expressed that ChatGPT helped them manage language challenges and time pressure. One student explained, “I use it only to check grammar and make my sentences sound more natural.” Another noted, “It saves time when I'm unsure about vocabulary or structure.” A few, however, acknowledged ethical tension, such as one who admitted, “Sometimes I know it's too much help, but it feels necessary to keep up”. These findings highlight a systematic perception–behavior gap in the ethical interpretation of AI use.

**Table 10:** Correlations between acceptability gaps and copy ratio for high-risk functions.

**Function**	**Pearson *r***	**Sig. (2-tailed)**	***N***	**Interpretation**
Drafting–major	0.49	< 0.001	60	Strong positive relationship
Translation	0.41	0.001	60	Moderate positive relationship
Paraphrasing	0.38	0.003	60	Moderate positive relationship

## 6 Discussion

This study explored how EFL students and teachers perceive, use, and evaluate ChatGPT in academic writing, integrating perceptual, behavioral, and ethical analyses. Drawing on SCT and Academic Integrity Theory, the discussion connects the findings from the four research questions with prior literature while emphasizing their theoretical implications and the integration of quantitative and qualitative evidence.

### 6.1 RQ1: actual behavioral use during controlled writing

Behavioral data revealed that learners spent approximately one-third of their writing sessions interacting with ChatGPT, mainly for editing (22 %), brainstorming (20 %), and drafting (16 %). Cluster analysis identified three distinct user types: constructive (42 %), hybrid (38 %), and substitutive (20 %). Constructive users primarily sought feedback and refinement, whereas substitutive users relied heavily on ChatGPT for major drafting and translation, producing the highest copy ratios (CR = 0.64).

These findings provide empirical support for Ibrahim and Kirkpatrick's (2024) distinction between constructive and substitutive uses, as identified in their systematic review, and align with the intervention results of (Khampusaen 2025) and (Mahapatra 2024), who reported that ethically integrated AI feedback enhanced writing fluency and confidence. Conversely, the substitutive group exemplifies the overreliance cautioned by (Lo et al. 2024) and (Alsaedi 2024), where extensive AI use compromises authorship and linguistic internalization.

From a sociocultural perspective, these patterns of use represent different positions along (Vygotsky 1978). Constructive users extended their ZPD by treating ChatGPT as scaffolding with a skilled peer helping them correct and develop ideas. Substitutive users, however, exemplified over-scaffolding (Baskara, 2023), where AI assistance took over their own cognitive effort. Interviews confirmed this pattern: many students used ChatGPT to improve language and refine their writing, saying, “I used it to polish sentences.” while others justified copying under pressure (“When time ran out, I copied a full paragraph”). Therefore, the tool's educational value depends more on how users think and act ethically than on its technical features. Importantly, qualitative evidence deepens this interpretation: while quantitative clustering identified behavioral types, interview data revealed the underlying motivations (e.g., efficiency, time pressure, linguistic insecurity) shaping these patterns (Hashemi and Babaii, 2013; Almanea, 2024).

### 6.2 RQ2: relationship between actual use and integrity perceptions

Correlational and regression analyses established a robust link between behavioral and ethical dimensions. Copy Ratio correlated negatively with integrity-belief scores (*r* = −0.46), while Levenshtein Distance—an indicator of revision—correlated positively (*r* = 0.42). Regression confirmed that higher copying predicted weaker ethical orientation, whereas revision effort and policy awareness predicted stronger ethical reasoning.

These results substantiate McCabe et al.'s (2001) and Bretag's (2013) theoretical claim that integrity is shown through actions, not just beliefs. The negative association between AI dependence and integrity supports Almanea's (2024) observation that students' self-reported attitudes may underrepresent misconduct. Many viewed partial reuse as “acceptable editing,” reflecting a moral gray zone similar to the ethical uncertainties (e.g., boundary between assistance and authorship) highlighted by (Cotton et al. 2024) in their discussion of AI use. The qualitative findings reinforce this interpretation by revealing how students rationalized reuse as “editing,” illustrating the cognitive conflict between perceived and enacted integrity. By contrast, students emphasizing revision viewed ChatGPT as a scaffold for noticing errors, aligning with Kim's (2025) assertion that guided AI interaction can promote metalinguistic awareness and self-regulation.

The mixed-methods evidence demonstrates a behavior–belief divergence: quantitative correlations identify the pattern, while qualitative narratives explain the ethical reasoning behind it. Therefore, this evidence highlights the need to build reflective AI literacy by teaching students how to use ChatGPT responsibly and justify their choices ethically. As (Lo et al. 2024) note, balancing efficiency with authenticity is vital to preserve the educational purpose of writing.

### 6.3 RQ3: perceptions of ChatGPT use and academic integrity

Findings revealed a distinct perception gap between students and teachers. Students viewed ChatGPT favorably as a helpful study aid (*M* = 3.87), while teachers had a more careful view (*M* = 3.25). This difference reflects earlier findings showing that students often view ChatGPT as a learning aid for improving grammar and vocabulary (Almanea, 2024; Xiao and Zhi, 2023), whereas teachers focus on risks to authenticity, fairness, and originality (Cotton et al., 2024; Werdiningsih and Marzuki Rusdin, 2024).

Regression analysis indicated that frequency of ChatGPT use, AI literacy, and policy awareness predicted students' integrity beliefs. Students with greater exposure and clearer understanding of institutional expectations reported stronger acceptance of responsible AI use. This pattern supports Bretag's (2013) argument that integrity depends not only on personal ethics but also on institutional clarity. Qualitative accounts further contextualize these results by showing that uncertainty about institutional policies often translated into situational ethical reasoning rather than deliberate misconduct. Many students were unsure about what university policy actually allows, showing how unclear communication can create ethical confusion. As (Alsaedi 2024) and (Hasan et al. 2025) suggest, integrity education and transparent guidance are prerequisites for ethical AI adoption in academia.

### 6.4 RQ4: perceptual and behavioral gaps between students and teachers

The mixed-design ANOVA confirmed that teachers applied stricter ethical standards across functions, particularly for drafting, translation, and paraphrasing. Students were more permissive toward these high-risk functions, viewing them as extensions of language support rather than violations. Kendall's W (0.48) indicated only moderate alignment between groups.

These gaps mirror earlier studies documenting teacher-student mismatches in AI ethics (Slamet, 2024; Bin-Nashwan et al., 2023; Almanea, 2024). Teachers emphasized fairness and authorship—reflecting an institutional integrity logic—while students emphasized linguistic and pragmatic benefits, a functional support logic. Through the integration of quantitative and qualitative evidence, statistical associations demonstrate the perception–behavior link, while interview data illuminate the contrasting moral logics underlying these differences. This confirms Zhai et al.'s (2024) claim that moral acceptance predicts behavioral substitution.

Qualitative evidence further illustrated these moral frameworks: teachers equated heavy ChatGPT use with plagiarism, while students saw it as compensation for linguistic disadvantage or time pressure. These differences highlight the need for shared ethical dialogue in EFL education that respects students' linguistic challenges while maintaining fairness.

Institutional ambiguity appears pivotal: where policies are vague, students rely on peer norms and personal judgment rather than institutional ethics (Hasan et al., 2025). From a theoretical standpoint [i.e., (Vygotsky 1978)], this highlights the intersection of sociocultural mediation and ethical framing: AI use emerges not only as a learning tool but as a negotiated social practice shaped by norms, pressures, and institutional discourse. Hence, promoting integrity in AI-integrated classrooms requires clear policies and teacher modeling of responsible use.

### 6.5 Pedagogical and ethical implications

Institutions should establish clear and context-specific AI policies that explicitly distinguish between constructive uses (e.g., brainstorming, outlining, editing) and high-risk uses (e.g., full drafting or translation), reflecting the behavioral patterns identified in this study (Almanea, 2024; Cotton et al., 2024). Such policies should include concrete examples of acceptable and unacceptable practices to reduce the uncertainty expressed by students in the qualitative findings.

Assessment design should shift from product-oriented evaluation toward process-based approaches. Incorporating staged drafting, revision tracking, and reflective commentaries can help instructors detect substitutive AI use while encouraging constructive engagement. For example, requiring students to submit drafts alongside short reflections on how AI tools were used may promote transparency and strengthen ethical awareness (Khampusaen, 2025).

At the pedagogical level, teachers should be trained to model scaffolded AI use aligned with sociocultural principles of guided learning. Explicit classroom demonstrations of responsible AI practices, such as using ChatGPT for feedback rather than text generation, can help students develop metacognitive awareness of ethical boundaries (Xiao and Zhi, 2023). Additionally, integrating AI literacy modules into writing courses can support students in critically evaluating when and how AI assistance is appropriate.

From a broader institutional perspective, alignment between policy, pedagogy, and assessment is essential. Without clear guidance, students may rely on peer norms or personal judgment, increasing perception–behavior mismatches. Establishing coordinated institutional frameworks that combine policy clarity, teacher training, and process-oriented assessment can support ethical AI integration while preserving the developmental goals of EFL writing (Cotton et al., 2024; Sullivan et al., 2023).

## 7 Conclusion

This study examined how EFL students use ChatGPT in academic writing and how observed practices align with integrity perceptions. The study makes three key contributions. First, it advances methodological approaches by integrating screen-recorded behavioral data with perceptual and qualitative evidence, addressing a major gap in prior self-report-driven research. Second, it provides theoretical insight into how sociocultural mediation and ethical decision-making intersect in AI-supported writing, showing that the educational value of GenAI depends on how learners regulate and interpret its use. Third, it offers practical implications for EFL pedagogy by highlighting the need for process-oriented assessment, AI literacy development, and clearer institutional guidance to support ethical AI integration.

This study has several limitations. The sample was restricted to female participants from a single Saudi institution, which may limit generalizability. The controlled laboratory design, while enabling behavioral observation, may not fully reflect naturalistic AI use. Additionally, the focus on short-term writing tasks limits conclusions about long-term language development. Future research should examine more diverse contexts, longitudinal learning outcomes, and larger qualitative datasets to better understand evolving AI practices in EFL writing.

Looking ahead, future studies could explore cross-cultural comparisons of AI ethics in language learning, longitudinal trajectories of AI-supported writing development, and discipline-specific variations in acceptable AI use. Expanding research to classroom ecologies and integrating discourse-analytic approaches may further illuminate how learners negotiate authorship, autonomy, and integrity in AI-mediated environments. As generative AI continues to reshape educational practices, sustained empirical and theoretical inquiry will be essential to ensure that technological innovation supports, rather than undermines, the developmental goals of language education.

## Data Availability

The original contributions presented in the study are included in the article/Supplementary material, further inquiries can be directed to the corresponding author.
