# Correction: Perceptions vs. practices: academic integrity and actual ChatGPT use among EFL students

**DOI:** 10.3389/fpsyg.2026.1868057

**Published:** 2026-05-11

**Authors:** Reem Alsadoon

**Affiliations:** English Department, Imam Mohammad Ibn Saud Islamic University (IMSIU), Riyadh, Saudi Arabia

**Keywords:** academic integrity, ChatGPT, EFL writing, perception, student behavior

Affiliation Imam Mohammad Ibn Saud Islamic University (IMSIU) was erroneously given as Imam Mohammad Ibn Saud Islamic University.

An incorrect number was provided for grant number IMSIU-DDRSP2502. The correct number is grant number IMSIU-DDRSP2602.

The original version of this article has been updated.

