# Supplementary material for: Perceptions vs. practices: academic integrity and actual ChatGPT use among EFL students
Source: Front Psychol. 2026 Mar 19;17:1796737. doi: 10.3389/fpsyg.2026.1796737 (PMC13044046; doi:10.3389/fpsyg.2026.1796737)
Supplement: Supplementary file 1 [file Supplementary_file_1.docx]

Supplementary Material

# Appendix A: Pre-task student questionnaire

**Purpose**
Before starting the writing task, please answer these short questions about your experience with AI tools (e.g., ChatGPT) and your views on acceptable use in academic writing.

## Background

English proficiency (self-rating)
  Beginner Elementary Intermediate Upper-Intermediate Advanced Near-native

Have you ever used any AI tool for learning/writing? No / Yes

AI tools used (check all): ChatGPT / Gemini / Copilot / Grammarly / QuillBot / Other ____

How often do you use AI tools for study tasks? Never / Monthly / Weekly / Daily

Have you received formal guidance on responsible AI use? Yes / No / Not sur

## Frequency of AI Use in Writing

Brainstorming: Never/Rarely/Sometimes/Often/Very Often

Outlining: Never/Rarely/Sometimes/Often/Very Often

Drafting (minor): Never/Rarely/Sometimes/Often/Very Often

Drafting (major): Never/Rarely/Sometimes/Often/Very Often

Editing: Never/Rarely/Sometimes/Often/Very Often

Translation: Never/Rarely/Sometimes/Often/Very Often

Paraphrasing: Never/Rarely/Sometimes/Often/Very Often

Summarizing: Never/Rarely/Sometimes/Often/Very Often

Citations: Never/Rarely/Sometimes/Often/Very Often

## Perceived Acceptability

*Scale: 1 = Completely unacceptable 5 = unacceptable*

Brainstorming: Completely unacceptable/ Unacceptable/Neutral/Acceptable/Completely acceptable

Outlining: Completely unacceptable/ Unacceptable/Neutral/Acceptable/Completely acceptable

Drafting (minor): Completely unacceptable/ Unacceptable/Neutral/Acceptable/Completely acceptable

Drafting (major): Completely unacceptable/ Unacceptable/Neutral/Acceptable/Completely acceptable

Editing: Completely unacceptable/ Unacceptable/Neutral/Acceptable/Completely acceptable

Translation: Completely unacceptable/ Unacceptable/Neutral/Acceptable/Completely acceptable

Paraphrasing: Completely unacceptable/ Unacceptable/Neutral/Acceptable/Completely acceptable

Summarizing: Completely unacceptable/ Unacceptable/Neutral/Acceptable/Completely acceptable

Citations: Completely unacceptable/ Unacceptable/Neutral/Acceptable/Completely acceptable

## Ethical Stance & Transparency

*Scale: 1 = Strongly disagree 5 = Strongly agree*
24. Submitting AI-generated paragraphs without disclosure is academic misconduct. Strongly disagree/ disagree/Neutral/Agree/ Strongly Agree
25. Using AI to generate ideas is acceptable if I write the essay myself. Strongly disagree/ disagree/Neutral/Agree/ Strongly Agree
26. If AI rewrites my paragraph, I should disclose that assistance. Strongly disagree/ disagree/Neutral/Agree/ Strongly Agree
27. Using AI to write most of the essay is unethical, even if I edit it slightly. Strongly disagree/ disagree/Neutral/Agree/ Strongly Agree
28. I feel confident I can use AI responsibly in academic writing. Strongly disagree/ disagree/Neutral/Agree/ Strongly Agree

## Policy Awareness

I understand my university’s policy on AI use. Strongly disagree/ disagree/Neutral/Agree/ Strongly Agree

What does your course policy require? No AI allowed/AI allowed with disclosure/AI freely allowed/I don’t know

## Pre-Task State

*Scale: 1 = Not at all 5 = Very*
32. I feel confident about completing the writing task. Not at all/Slightly/Moderately/Very/Very Much
33. I feel nervous about the writing task. Not at all/Slightly/Moderately/Very/Very Much
34. I expect to use AI during the task. Not at all/Slightly/Moderately/Very/Very Much
35. I intend to follow my own integrity standards during the task. Not at all/Slightly/Moderately/Very/Very Much

# Appendix B: Post-task student questionnaire

**Purpose:**
Please complete this short survey after finishing your writing task. Your responses are anonymous and will not affect your grade.

## ****Reported Use During the Task****

Did you use **ChatGPT** during the writing task?
 □ Yes □ No

How many times did you open ChatGPT during the task?
 □ 1–2 □ 3–5 □ 6–10 □ More than 10

Approximately how many minutes did you spend using ChatGPT?
 □ <5 □ 5–10 □ 11–20 □ 21–30 □ More than 30

Which functions did you use ChatGPT for? (check all that apply)
 □ Brainstorming ideas (BRAIN)
 □ Making an outline (OUTL)
 □ Drafting short paragraphs (DRAFT-minor)
 □ Drafting large sections/full essay (DRAFT-major)
 □ Editing/polishing my text (EDIT)
 □ Translation (TRANS)
 □ Paraphrasing (PARA)
 □ Summarizing (SUMM)
 □ Creating citations/references (CITE)

Which single function did you rely on the most?

 □ Brainstorming ideas (BRAIN)
 □ Making an outline (OUTL)
 □ Drafting short paragraphs (DRAFT-minor)
 □ Drafting large sections/full essay (DRAFT-major)
 □ Editing/polishing my text (EDIT)
 □ Translation (TRANS)
 □ Paraphrasing (PARA)
 □ Summarizing (SUMM)
 □ Creating citations/references (CITE)

## ****Self-Evaluation of ChatGPT Use****

Scale 1 = Strongly disagree 5 = Strongly agree

ChatGPT helped me generate better ideas. Strongly disagree/ disagree/Neutral/Agree/ Strongly Agree

ChatGPT improved my grammar and vocabulary. Strongly disagree/ disagree/Neutral/Agree/ Strongly Agree

ChatGPT made it easier to organize my essay. Strongly disagree/ disagree/Neutral/Agree/ Strongly Agree

I spent less time writing because of ChatGPT. Strongly disagree/ disagree/Neutral/Agree/ Strongly Agree

ChatGPT’s feedback was useful and reliable. Strongly disagree/ disagree/Neutral/Agree/ Strongly Agree

I edited or rewrote most of the text generated by ChatGPT. Strongly disagree/ disagree/Neutral/Agree/ Strongly Agree

My essay reflects my own voice and understanding. Strongly disagree/ disagree/Neutral/Agree/ Strongly Agree

Using ChatGPT in this task was ethically acceptable. Strongly disagree/ disagree/Neutral/Agree/ Strongly Agree

## ****Perceived Integrity and Policy Awareness****

Scale 1 = Strongly disagree 5 = Strongly agree

I was careful to use ChatGPT within ethical limits. Strongly disagree/ disagree/Neutral/Agree/ Strongly Agree

I would disclose ChatGPT use if required by my instructor. Strongly disagree/ disagree/Neutral/Agree/ Strongly Agree

My writing today meets academic-integrity standards. Strongly disagree/ disagree/Neutral/Agree/ Strongly Agree

I understand what counts as misconduct when using ChatGPT. Strongly disagree/ disagree/Neutral/Agree/ Strongly Agree

My instructors’ expectations about ChatGPT use are clear and fair. Strongly disagree/ disagree/Neutral/Agree/ Strongly Agree

## ****Post-Task Reflections****

Compared with your expectations before the task, did you use ChatGPT…
 □ Less than expected □ About the same □ More than expected

How helpful was ChatGPT overall for this task? Not helpful/Somewhat unhelpful/Neutral/ Helpful/ Extremely helpful

Would you use ChatGPT again for similar academic tasks?
 □ Definitely not □ Probably not □ Maybe □ Probably □ Definitely

## ****Post-Task State****

Scale 1 = Not at all 5 = Very

I feel satisfied with my essay. Not at all/Slightly/Moderately/Very/Very Much

I learned something new about writing today. Not at all/Slightly/Moderately/Very/Very Much

I feel confident I can use ChatGPT responsibly in future assignments. Not at all/Slightly/Moderately/Very/Very Much

# Appendix C: Teacher questionnaire

**Purpose:**
This survey explores EFL teachers’ perceptions of ChatGPT use in academic writing, their beliefs about acceptable and unacceptable practices, and their awareness of institutional policy. All responses are anonymous.

## ****Background Information****

Years of EFL teaching experience
 □ 1–3 □ 4–7 □ 8–12 □ 13–20 □ More than 20

Have you personally used ChatGPT for teaching or academic purposes?
 □ Yes □ No

If yes, how often do you use ChatGPT?
 □ Rarely □ Monthly □ Weekly □ Daily

## ****Perceptions of ChatGPT Functions in Writing****

Scale: 1 = Completely unacceptable 5 = Completely acceptable

Teachers’ views on **how acceptable** it is for students to use ChatGPT for:

Brainstorming ideas (BRAIN) Completely unacceptable/ Unacceptable/Neutral/Acceptable/Completely acceptable

Making an outline (OUTL) Completely unacceptable/ Unacceptable/Neutral/Acceptable/Completely acceptable

Drafting short paragraphs (DRAFT-minor) Completely unacceptable/ Unacceptable/Neutral/Acceptable/Completely acceptable

Drafting large sections or a full essay (DRAFT-major) Completely unacceptable/ Unacceptable/Neutral/Acceptable/Completely acceptable

Editing or polishing grammar and vocabulary (EDIT) Completely unacceptable/ Unacceptable/Neutral/Acceptable/Completely acceptable

Translation between Arabic and English (TRANS) Completely unacceptable/ Unacceptable/Neutral/Acceptable/Completely acceptable

Paraphrasing source text (PARA) Completely unacceptable/ Unacceptable/Neutral/Acceptable/Completely acceptable

Summarizing articles or readings (SUMM) Completely unacceptable/ Unacceptable/Neutral/Acceptable/Completely acceptable

Creating citations and references (CITE) Completely unacceptable/ Unacceptable/Neutral/Acceptable/Completely acceptable

## ****Perceptions of Integrity and Pedagogical Impact****

Scale: 1 = Strongly disagree 5 = Strongly agree

ChatGPT poses serious risks to academic integrity. Strongly disagree/ disagree/Neutral/Agree/ Strongly Agree

ChatGPT can enhance students’ writing if used responsibly. Strongly disagree/ disagree/Neutral/Agree/ Strongly Agree

Most students over-rely on ChatGPT instead of improving their own skills. Strongly disagree/ disagree/Neutral/Agree/ Strongly Agree

Students should disclose any ChatGPT assistance in their writing. Strongly disagree/ disagree/Neutral/Agree/ Strongly Agree

Teachers should design assessments that minimize misuse of ChatGPT. Strongly disagree/ disagree/Neutral/Agree/ Strongly Agree

ChatGPT helps reduce grading workload or provides useful feedback models. Strongly disagree/ disagree/Neutral/Agree/ Strongly Agree

ChatGPT use promotes critical thinking and reflection when guided properly. Strongly disagree/ disagree/Neutral/Agree/ Strongly Agree

Current institutional rules about AI use are clear and sufficient. Strongly disagree/ disagree/Neutral/Agree/ Strongly Agree

I feel confident explaining to students what constitutes ethical ChatGPT use. Strongly disagree/ disagree/Neutral/Agree/ Strongly Agree

## ****Policy and Instructional Practices****

Does your institution have a written policy on AI or ChatGPT use?
 □ Yes □ No □ Not sure

Have you discussed AI ethics or ChatGPT guidelines with your students this semester?
 □ Yes □ No

How strict should institutional penalties be for undisclosed ChatGPT use?
 □ Very lenient □ Somewhat lenient □ Moderate □ Strict □ Very strict

# Appendix D: Coding Manual: ChatGPT Prompt Functions

**Purpose**
This manual explains how to use the coding sheet to classify each student prompt given to ChatGPT.

## General Rules

Read the full Prompt_Text.

Decide which function(s) the prompt serves.

Mark “1” in the relevant column(s); leave other columns blank.

If the prompt serves more than one function, mark multiple codes.

Use the Comments column for unclear or mixed cases.

## Code Definitions & Examples

BRAIN (Brainstorming)

Prompts asking for ideas, topics, or examples.

*Example:* “Give me 5 ideas for an essay about technology.”

OUTL (Outlining)

Prompts requesting structure, bullet points, or organization.

*Example:* “Make me a 3-paragraph outline for climate change.”

DRAFT_minor (Small Draft)

Prompts asking for a short passage (sentence or paragraph).

*Example:* “Write a 70-word conclusion about allowing children to have cell phones.”

DRAFT_major (Large Draft)

Prompts asking for a long section or full essay.

*Example:* “Write a 350-word essay on gaming and second language learning with references.”

EDIT (Editing / Polishing)

Prompts requesting grammar correction, style improvement, or academic tone.

*Example:* “Fix the grammar in this paragraph.”

TRANS (Translation)

Prompts requesting translation between languages.

*Example:* “Translate this Arabic sentence into English.”

PARA (Paraphrasing)

Prompts requesting rewriting to avoid copying.

*Example:* “Paraphrase this paragraph in different words.”

SUMM (Summarizing)

Prompts requesting condensation of a long text.

*Example:* “Summarize this article in 3 sentences.”

CITE (Citation / Referencing)

Prompts requesting references or citation formatting.

*Example:* “Create an APA reference for this website.”

## Ambiguity Rules

If a prompt combines two clear functions (e.g., “Translate and paraphrase”), mark both codes.

If unclear, use the Comments column to explain your decision.

Always lean toward the main purpose of the prompt.

## Reliability

Two coders should independently code the same sample (10–15%).

Comparing results, aiming for κ ≥ .75 agreement.

Discussing differences and adjusting before coding the full dataset.

# Appendix E: R Script for Text Comparison Metrics

# ---------------------------------------------------------

# R Script: Calculating Copy Ratio and Levenshtein Distance

# ---------------------------------------------------------

# library(stringdist)

library(quanteda)

library(readr)

library(dplyr)

# -------------------------------

# 1. Load Data

# -------------------------------

# "chatgpt_text" and "student_text" for each participant

# Example: data.csv

data <- read_csv("data.csv")

# -------------------------------

# 2. Compute Metrics

# -------------------------------

# Levenshtein Edit Distance (normalized)

data <- data %>%

mutate(

edit_distance = stringdist(chatgpt_text, student_text, method = "lv"),

normalized_edit = edit_distance / nchar(student_text)

)

# Copy Ratio (Cosine Similarity based on token overlap)

# Create document-feature matrix (DFM)

dfm_data <- dfm(corpus(c(data$chatgpt_text, data$student_text)))

# Compute cosine similarities in pairs

similarities <- textstat_simil(dfm_data, method = "cosine")

# Extract every second row (ChatGPT vs. Student text pairs)

# data$copy_ratio <- diag(as.matrix(similarities)[1:(nrow(data)), (nrow(data)+1):(2*nrow(data))])

# -------------------------------

# 3. Export Results

# -------------------------------

write_csv(data %>% select(chatgpt_text, student_text, normalized_edit, copy_ratio),

"text_comparison_results.csv")

# -------------------------------

# 4. Interpretation Guide

# -------------------------------

# normalized_edit → smaller = closer to ChatGPT (less editing)

# copy_ratio → larger = higher similarity (more direct copying)

# import 'text_comparison_results.csv' into SPSS.

# ---------------------------------------------------------

# Appendix F: Semi-structured interview

## Opening

Can you briefly describe how you felt during the writing task?

Before the task, had you used ChatGPT for academic writing? If yes, in what ways?

## Experience during the writing task

How did you decide when and how to use ChatGPT during the writing task?

Which ChatGPT functions did you find most useful today (e.g., brainstorming, drafting, editing)? Why?

Did you make any changes to ChatGPT’s suggestions before adding them to your essay? Can you give an example?

## Perceptions of Integrity

When using ChatGPT, how do you decide what is acceptable and what might be considered misconduct?

Some people say using ChatGPT for writing is “cheating,” while others call it a “learning support.” What is your view?

Did you feel any conflict between wanting to improve your writing and wanting to stay within ethical limits?

## Reflection and Future Intentions

What benefits and challenges do you see in using ChatGPT for writing in the future?

If your instructor asked you to disclose ChatGPT use, how would you feel about that?

Overall, what did you learn from this experience about writing and academic integrity?

## Closing

Is there anything else you’d like to share about your experience with ChatGPT during this task?

# Appendix G: Consent Form

**Title of the Study**
Perceptions vs. Practices: Academic Integrity and Actual ChatGPT Use among EFL Students

**Principal Investigator**
Reem Alsadoon, Associate Professor of Linguistics
[English department / College of Languages and Translation]
[Imam Mohammed Ibn Saud Islamic University]
Email: [raalsadoon@imamu.edu.sa]

You are invited to participate in a research study examining how EFL students use ChatGPT in academic writing and how this relates to academic integrity. Participation is voluntary. If you agree, you will complete a timed writing task in a computer lab where ChatGPT use is permitted, allow screen recording of your on-screen activity during the task, and complete short questionnaires; some participants may also take part in a brief interview. The study involves minimal risk, and you may withdraw at any time without penalty or academic consequences. All data will be kept confidential, anonymized, securely stored, and used only for research purposes. By participating, you confirm that you understand the study and voluntarily consent to take part.
